# The size and shape of parasitic larvae of naiads (Unionidae) are not dependent on female size

**DOI:** 10.1038/s41598-021-03143-9

**Published:** 2021-12-09

**Authors:** Adam M. Ćmiel, Jacek Dołęga, David C. Aldridge, Anna Lipińska, Feng Tang, Katarzyna Zając, Manuel Lopes-Lima, Tadeusz Zając

**Affiliations:** 1 Institute of Nature Conservation, Polish Academy of Sciences, al. A. Mickiewicza 33, 31-120 Kraków, Poland; 2 Department of Zoology, The David Attenborough Building, University of Cambridge, Pembroke Street, Cambridge, CB2 3QZ UK; 3 CIBIO/InBIO-Research Center in Biodiversity and Genetic Resources, University of Porto, Campus Agrário de Vairão, 4485-661 Vairão, Portugal

**Keywords:** Ecology, Evolutionary ecology

## Abstract

The naiads, large freshwater mussels (Unionida), have very long life spans, are large-bodied, and produce thousands to millions of larvae (glochidia) which typically must attach to host fish tissues to metamorphose into a juvenile mussel. Glochidia develop within a female's marsupial gill demibranch, thus their number is restricted by female size. However, larger mussels acquire more energy, which could be invested in either larger-sized glochidia, in a more glochidia, or a combination of both. The high level of host specialization seen in many naiads may constrain glochidial size and shape around a narrow optimum, while naiads that use a wide range of host fishes may be predicted to possess greater plasticity in glochidial morphology. In this paper, we investigated the relationship between maternal body size and progeny body size and shape, aided by modern digital microscopy. We analyzed the between- and within- species variation of glochidia size and shape relative to female size in four widespread species of European naiads: *Anodonta anatina*, *Anodonta cygnea*, *Unio crassus* and *Unio tumidus*. Whereas the total reproductive output is collinear with female body size, substantial differences between species in glochidia size were found within genus *Anodonta*, but not genus *Unio* where glochidial size is remarkably consistent. The glochidial shape, however, differed within both *Unio* and *Anodonta*. We interpret this constant within-species glochidial size in *Unio* as reflecting a constraint imposed by the likelihood of successful transmission onto and off from a narrow range of hosts, whereas their shape seems to be less constrained. The *Anodonta* species, inhabiting a wide spectrum of habitats and using more than twice the number of fish hosts than *Unio* spp., have larger glochidia with greater variation in size and shape. Our results suggest that measures of glochidial variability may also serve as an indicator of host specificity in other naiads.

## Introduction

Parental investment into offspring is often set against a framework of r- and K-strategies^1^, forming a continuum, bounded by two end-point strategies: an r-strategist would be characterised by the production of many small offspring, which mature early, have a short life expectancy, and a high mortality rate, whilst a K-strategist would invest in few large offspring, with a slow maturation rate, a relatively high survival rate and are long-lived. However, in most systems the observed variation in life-history strategies is too complex to be explained by an r–K continuum alone^2^. This complexity is exemplified in the naiads, large freshwater mussels (Unionida), which are among the most threatened animal groups on the planet^3,4^ and whose biology remains poorly studied^5^. Intriguingly, the life history and reproductive strategy of naiads reflect a combination of traits from both r- and K-strategies: they produce many offspring of microscopic size with low investment in each individual, yet adults are relatively large (e.g. Ref.^6^), with some species only reaching maturity over 30 years^7^, and a life expectancy of more than 100 years^6,8^ with individuals reproducing many times through their lifetime.

The enormous fecundity of naiads (ranging from thousands to millions of eggs per female^9^) is strongly related to body size^10–12^, both within and among species, and fecundity typically increases with female shell length^13^. However, life-history traits in Unionidae are highly variable within and among species, and very little is known about energy allocation in reproduction and related trade-offs (e.g. number vs size of the offspring in relation to parent growth vs reproduction). Indeed, some research suggests that developing glochidia are supplied by the female with calcium carbonate for shell production^14–17^, which may indicate an additional investment of females into their offspring. This group provides a particularly interesting opportunity for the analysis of life-history trade-offs: naiads differ from most other organisms in their possession of an obligatory parasitic stage of their larvae, which is necessary to complete their ontogenic development^18^. To complete the life cycle the larva (glochidium), after being expelled by the female into the water column, must typically attach to the gills or fins of a host fish^19–21^. However, fewer than one in 1 million find a suitable host and survive^22–24^, suggesting that selection will act strongly towards optimised infestation efficiency and driving a very narrow variation around optimal glochidial phenotypes.The degree of host specificity varies among species of naiads from specialists able to successfully parasitize only one or a few closely related fish species to generalists which can complete development on a taxonomically wide range of fish species^25^. Host specialism/generalism is likely to further drive trade-offs in glochidial investment, with selection potentially favouring narrower phenotypic variability in the glochidia of specialists than in generalists.

The probability of fish infestation will determine population-level recruitment dynamics^26^, and the infestation probability should depend on frequencies of the encounter between larva and fish host which in turn should depend on the local density of both glochidia and fish. The glochidium is equipped with a larval thread (Ref.^7^ and citations therein) which is assumed to enable it to float in the water or attach to roots and aquatic vegetation, or the gills or fins of its fish host. The low investment in individual glochidia is reflected by their low flesh content^27^, simple morphology^9^, and microscopic size (from 0.05 to 0.45 mm^12^).

The brooding capacity of a female, and thus the maximum number of glochidia in a brood, should be directly related to the volume of marsupia, which in turn is somewhat constrained by shell volume. Because infestation probability is determined by the encounter rate with fish, species that broadcast free glochidia typically possess extraordinarily high fecundity at the cost of reduced glochidial size to maximize the probability of host infection^7^. This generates selection on the optimal size of glochidia, balancing efficiency of successful infestation upon fish against maximal propagule number. On the other hand, there is some evidence of glochidia size being larger than expected in nutrient-rich environments^28^, which may enable them to live longer, thus increasing the likelihood of fish infestation; suggesting that additional resources may mediate the trade-offs between propagule number and size.

A further component of glochidium morphology that will be subject to selection is their shape, which is expected to influence infestation probability^29^. Glochidium shape and morphology have been shown to vary depending on the location of likely attachment upon on the host fish. For example, glochidia of species that target host gills are typically small and rounded, whilst those that encapsulate scales and fins typically possess large hooks^19^.

In this paper, we investigated the relationships between shell length of the female, glochidia size (length), glochidia shape and glochidia number in four species of European Naiads; two of the genus *Anodonta* (*Anodonta anatina*, *Anodonta cygnea)* and two of the genus *Unio* (*Unio crassus* and *Unio tumidus*). We aimed to fill the gap in knowledge about this important life-history stage of naiads by answering three key questions: (i) Do large freshwater mussel females invest more in individual progeny size than do small females? (ii) Is glochidium size and shape plastic or highly constrained between populations of the same mussel species? (iii) Do mussel species with a wider range of host fishes (i.e. *Anodonta* spp.) show greater variability in glochidia size and shape than those species that use a narrow host range (i.e. *Unio* spp.)?

## Materials and methods

### Mussel collection, glochidia and marsupia measurements

Wild mussels used in the study were collected using bottom-scrapers (rake with netting) in lakes and by wading with bathyscopes in rivers. Presence of glochidia was identified in each captured mussel by the gentle opening of the shell valves and visual inspection of the marsupia. Maturity of glochidia used in analyses was confirmed by evaluation of their morphology^30^ and exposing a subsample (approx. 30 ind.) to a salt (NaCl) solution; closure of valves indicated glochidia were mature and alive^31^, after which the glochidia were preserved in 95% ethanol. Only gravid females with full marsupia and fully developed glochidia were used for collecting samples. Four species of naiads were used: 33 females of *Anodonta cygnea* were collected on 26th and 31st March 2015 from Zalew Pińczowski reservoir near the town of Pińczów (central Poland; 50° 31′ 03.57″ N, 20° 31′ 03.57″ E); 37 individuals of *Anodonta anatina* were collected on 22nd January 2020 from Zesławice *No. 2* reservoir near Kraków (southern Poland; 50° 06′ 30.9″ N, 20° 01′ 51.2″ E); 24 individuals of *Unio tumidus* were collected on 8th May 2019 from the river Belnianka near Marzysz (central Poland; 50° 46′ 41.09″ N, 20° 42′ 28.97″ E); and 15 individuals of *Unio crassus* were collected on the 2nd and 19th June 2018 from the river San near Procisne (south-east of Poland; 49° 11′ 50.86″ N, 22° 40′ 56.89″ E). To enable within-species comparisons between different populations, gravid *U. crassus* females were collected from two additional populations: 11 individuals at vicinity of Lubaszowa in June 2018 from the river Biała (south of Poland; 49° 51′ 37″ N, 21° 01′ 59″ E), and five individuals at Leńcze from the river Cedron (south of Poland; 49° 53′ 17″ N, 19° 44′ 04″ E). For *U. crassus* the rivers differed in habitat characteristics: while the river San is a typical mountainous river with a rocky bottom, the river Biała is a typical river of foothills, with a pool-riffle system dominated by gravel. The river Cedron is a lowland meandering river dominated by fine sediments and with fine gravel present only in riffles. The Zalew Pińczowski reservoir and the Zasławice reservoir are seminatural, eutrophic, shallow water bodies similar to each other in general ecological characteristics. The river Belnianka is a small lowland meandering river with a sandy bottom.

**Figure 1:**
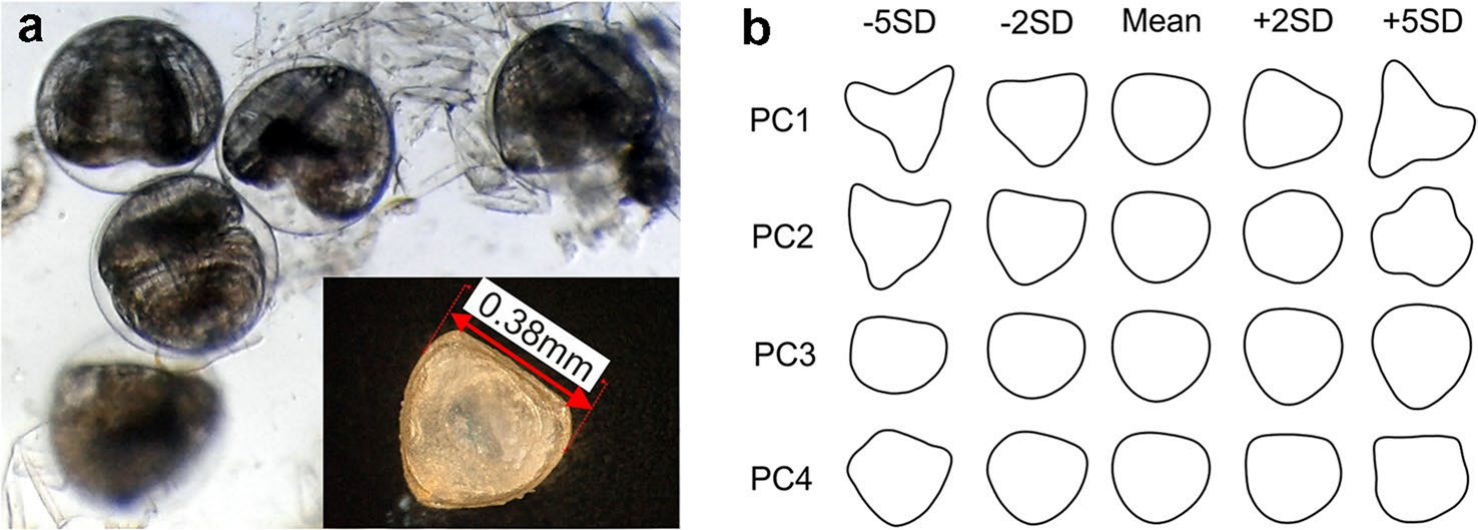
(**a**) Measurement of the length of flat-positioned glochidia of *A. anatina*; in the background almost fully grown glochidia of *U. crassus* still enclosed within the egg membranes. (**b**) Reconstructed closed contour of glochidia (mean, mean ± 2 SD and mean ± 5 SD) visualizing the shape aspect described by the significant Principal Components obtained from Elliptical Fourier Descriptors.

Glochidia of each species were extracted from marsupia by gently shaking the marsupium content in water in a Petri dish. Released glochidia were measured under a Keyece VHX-950F microscope equipped with the software ‘Find objects’ and ‘Measurement’ functions, which allowed precise measurement of each glochidium. Only flat-positioned glochidia with completely visible valves were selected for measurement (Fig. 1a). Because in previously published papers on glochidia size the definition of ‘length’ and ‘width’ of the shell is interchanged, for this study we define the glochidium valve length accordingly to the measurements of adults, i.e. the valve length is defined as the longest axis of the valve (the largest distance) measured along the line parallel to shell hinge (Fig. 1a). A sample of 30 randomly selected glochidia was measured for each gravid female.

To obtain a wet mass of marsupia, gravid females with full marsupia were collected for the purpose of the other studies (*A. cygnea* in 2003, *U. crassus* in Cedron in 2011, all other samples in 2018). They were taken from the water and transported to the laboratory, where they were sacrificed and immediately dissected: full marsupia were cut off the body using surgical scissors and weighed using an electronic laboratory scale (RADWAG WLY 0.6/1.2/D2; precision 0.1 g).

The effect of the preservation of glochidia in 95% ethanol was tested on glochidia taken from the gravid *A. anatina*. This species produces the largest glochidia among analyzed species, maximising the potential for detecting any influence of alcohol preservation on glochidia size. Fifteen fresh glochidia were taken from marsupia of living mussels and immediately measured. Then, each glochidium was stored in a separate numbered plastic Eppendorf tube filled with 95% ethanol and measured again after 72 h.

This research was conducted in full compliance with the ethical codes and legislation of the Republic of Poland. Collection, extraction of glochidia from marsupia, and killing of protected mussel species (*A. cygnea* and *U. crassus*) individuals were performed under the necessary permits: DZP-WG.6401.01.26.2018.bp (*U. crassus* in River San), DZP-WG.6401.01.31.2018.bp (*U. crassus* in River Biała), DOP-OZGIZ.6401.01.106.2011.ls (*U. crassus* in River Cedron), WPN.I.6401.56.2015.AC and DOPweg-4201-01-12/03/jr (*A. cygnea* in Zalew Pińczowski reservoir).

### Statistical analysis

All statistical analyses were performed using Statistica 13.1 software (Dell Inc.).

### Glochidia size analyses

Because the homogeneity of variance assumption was not fulfilled, the differences in mean glochidia size between species were analysed using nonparametric Kruskal–Wallis and multiple comparisons post-hoc tests. The relationship between the wet weight of marsupium and female shell length was analyzed using Pearson *r* correlations. The relationship between female shell length and mean length of glochidia was analyzed by fitting a linear function *g* = *s *× *l* + *c*, where *g* is a mean glochidium length, *s* is a slope coefficient, *l* is female shell length and *c* is a constant.

To test the influence of female body size with the controlled influence of species, variability within given female, and interactions between the analysed factors, Mixed-design General Linear Models were constructed. The mixed design is used when there is at least one within-subjects factor and at least one between-subjects factor in the same dataset. The constructed Mixed-design GLM scheme was: length of each measured glochidium (30 continuous dependent variables), species (between-subject categorical factor), female length (between-subject continuous predictor), within subject effect (WSE, which represents the variability of glochidia length within females), and the interactions between them (species × female length, WSE × species, WSE × species × female length). From this, we analysed the influence of the variability in glochidia length within each analyzed female itself (WSE). Mixed-design GLMs were also used to analyse if variability in glochidia length within each female depends on female length (WSE × female length), depends on mussel species (WSE × species) or depends both on female length and mussel species (WSE × female length × species), which is the main advantage of this approach over analysing separately the relationship between female length and lengths of its glochidia, and analyzing differences in glochidia length between species.

In the first Mixed design GLM model, the data for *U. crassus* were pooled across the three rivers. To analyze if the influence of female length on glochidia size in *U.crassus* differed between habitats (rivers), the same Mixed design GLM model was constructed but categorical factor "species" replaced by categorical factor: "river".

To determine the significance of glochidium size between species that might be used for species determination, we applied discriminant function analysis, using measured glochidium length data.

### Glochidia shape analysis

Shape of glochidia was analyzed using Elliptical Fourier descriptors (EFDs) with SHAPE 1.3 software^32^. After noise reduction, the closed contours of glochidia were chain-coded^33^. The EFD coefficients were calculated by discrete Fourier transformation of the chain-coded contours following the procedures given by Kuhl and Giardina^34^. EFDs were normalized with a procedure based on the first harmonic ellipse that corresponds to the first Fourier approximation to the contour information. Glochidia shape was approximated by the first twenty harmonics, which leads to a large number of normalized EFD coefficients. Thus, to summarize the information contained in them, we performed Principal Component Analyses (PCA) based on variance–covariance matrices. The homogeneity of variance of each principal component score between mussel species was tested with Levene’s tests and because the homogeneity of variance assumption was not fulfilled, the differences in PC scores between species were analysed using nonparametric Kruskal–Wallis and multiple comparisons *post-hoc* tests. The effect that principal components described for shell shape was visualized in relation to the mean effect by inverse recalculation of EFDs using an eigenvector matrix. Only significant PCs were visualised, with extreme morphologies visualised for mean ± 2 SD and mean ± 5 SD). This enabled visualization of the shape aspect described by the given PC.

## Results

In total, 3780 glochidia from 126 females were measured. Sizes of female mussels were [all measurements are expressed in mm]: *A. anatina* (N = 37, mean length = 120, SD = 13, range 73–140); *A. cygnea* (N = 33, mean = 113, SD = 18, range 84–146); *U. tumidus* (N = 24, mean = 91, SD = 16, range 51–116); *U. crassus* from the river Biała (N = 11, mean = 64, SD = 5, range 54–69); *U. crassus* from the river Cedron (N = 5, mean = 58, SD = 3, range 56–63); *U. crassus* from the river San (N = 15, mean = 47, SD = 9, range 36–62). Measurement of the length of flat-positioned glochidium was presented at Fig. 1a.

**Figure 2:**
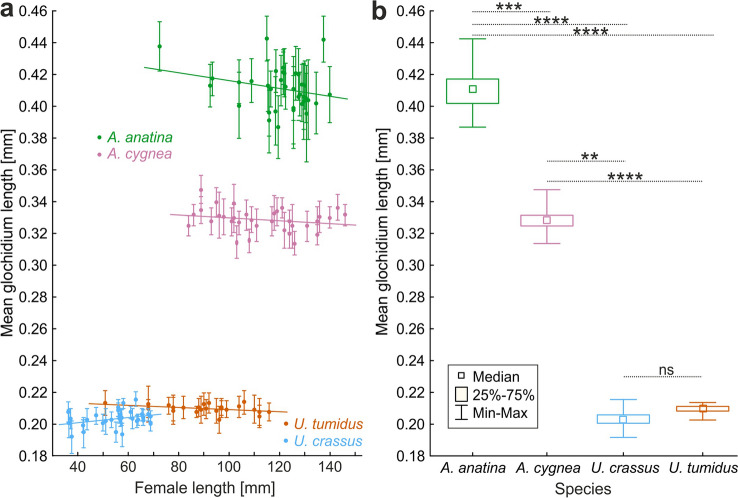
(**a**) The relationship between female shell length and mean length of its glochidia (mean ± SD). (**b**) Box plots of the differences in mean glochidia size between species with the results of post-hoc tests of differences between species. *ns* not significant, *p < 0.05, **p < 0.01, ***p < 0.001, ****p < 0.0001.

Descriptive statistics for measured glochidia with comparison to the data from other studies are presented in Table 1. The glochidia size varied significantly between species (Fig. 2a,b). Mean glochidium length was significantly different between species (Kruskal–Wallis test; H = 113.3; df = 3; p < 0.0001). Post-hoc tests (multiple comparisons) showed that the differences were significant between every pair of analysed species, except for *U. crassus* and *U. tumidus* (Fig. 2b).

**Table 1:** Descriptive statistics of glochidia for each species measured during this study in comparison to other measurements of the same species. *N* sample size, *SD* standard deviation, *Min.* minimum, *Max.* maximum, range refers to the maximum difference recorded in all presented data, *range/mean size* range of the glochidium length variability standardized by the averaged mean sizes presented in the table, (–) not available.

Species	Site	N	Mean [mm]	SD	Min [mm]	Max [mm]	Range min.–max [mm]	Range/mean size	Source
*Anodonta anatina*		30	0.36	–	–	–	–	–	^35^
		–	–	–	0.35	0.36	0.01	–	^36^
		–	0.36	–	–	–	–	–	^37^
		–	0.35	–	–	–	–	–	^38^
		–	0.36	–	–	–	–	–	^39^
		–	0.50	0.08	0.45	0.57	0.12	0.24	^28^
		–	0.34	0.02	0.34	0.42	0.08	0.24	^28^
		–	–	–	0.27	0.57	0.3	–	^40,41^
		–	–	–	0.33	0.38	0.05	–	^42^
		30	0.36	0.02	–	–	–	–	^43^
		–	0.35	–	–	–	–	–	^44^
		–	0.34	–	–	–	–	–	^45^
		7	0.36	0.04	0.35	0.36	0.01	0.03	^46^
		1110	0.41	0.02	0.31	0.47	0.16	0.39	This study
	Mean	∑ = 1177	0.37	0.04	0.36	0.46	0.1	0.27	
*Anodonta cygnea*		30	0.32	–	–	–	–	–	^35^
		–	0.35	–	–	–	–	–	^47^
		–	–	–	0.27	0.36	0.09	–	^40,41^
		2	0.32	0.05	0.31	0.34	0.03	0.09	^48^
		–	0.32	–	–	–	–	–	^44^
		–	0.31	–	–	–	–	–	^45^
		–	0.34	–	–	–	–	–	^49^
		9	0.35	0.04	0.34	0.36	0.02	0.06	^50^
		990	0.33	0.01	0.25	0.37	0.12	0.36	This study
	Mean	∑ = 1031	0.33	0.03	0.31	0.38	0.07	0.21	
*Unio crassus*		–	–	–	0.18	0.23	0.05	–	^40,41^
			0.22				–	–	^45^
	PL: Biała	330	0.20	0.004	0.19	0.22	0.03	0.15	This study
	PL: San	450	0.20	0.010	0.17	0.23	0.06	0.30	This study
	PL: Cedron	150	0.21	0.005	0.20	0.23	0.03	0.14	This study
	PL: pulled	930	0.20	0.008	0.17	0.23	0.06	0.30	This study
	Mean	∑ = 930	0.21	0.006	0.19	0.23	0.04	0.19	
*Unio tumidus*		–	–	–	0.19	0.22	0.03	–	^40,41^
		–	0.20	–	–	–	–	–	^50^
		–	0.21	–	–	–	–	–	^45^
		720	0.21	0.007	0.18	0.25	0.07	0.33	This study
	Mean	∑ = 720	0.21	0.007	0.19	0.24	0.05	0.24

Preservation of glochidia in ethanol had no effect on glochidia size (mean length of fresh glochidia 0.372 mm; mean length of alcohol preserved glochidia 0.373 mm; paired t = − 1.1, df = 13, p = 0.3087).

The marsupium wet weight for all studied species was highly and significantly correlated with female shell length (Pearson correlations; *A. anatina*: r = 0.80, N = 21; *A. cygnea*: r = 0.84, N = 58; *U. tumidus*: r = 0.91, N = 38; *U. crassus*: r = 0.79, N = 35; all correlations are significant at p < 0.0001).

Fitting linear functions describing the relationship between female shell length and mean length of its glochidia for each species separately showed that the slope of regression line was not significantly different from zero in all analyzed species (*A. anatina*: R^2^ = 0.26, slope = − 0.0002, t = − 1.6, p = 0.123; *A.cygnea*: R^2^ = 0.22, slope = − 0.00009, t = − 1.3, p = 0.220; *U. crassus*: R^2^ = 0.34, slope = 0.0002, t = 2.0, p = 0.550; *U. tumidus*: R^2^ = 0.35, slope = − 0.00005, t = − 1.8, p = 0.089; Fig. 2a).

The models controlling for other factors (Mixed design GLMs) showed that the only significant factor influencing the size of glochidia was species. All other predictors, including variability of glochidium length within a particular female (WSE), and all interaction terms were not significant (Table 2; Fig. 2a).

**Table 2:** The results of Mixed-design GLMs of the influence of female shell length on glochidia length. *WSE* within subject effect, *SS* sum of squares, *df* degrees of freedom, *MS* mean square.

Model	Effect	SS	df	MS	F	p
I (all species)	Intercept	7.0	1	7.0	3401.0	< 0.0001
	Species	0.7	3	0.2	117.1	< 0.0001
	Female length	0.002	1	0.002	1.1	0.2881
	Species × female length	0.01	3	0.004	1.8	0.1591
	WSE	0.005	29	0.0002	1.4	0.0610
	WSE × species	0.009	87	0.0001	0.8	0.9138
	WSE × female length	0.004	29	0.0001	1.1	0.3851
	WSE × species × female length	0.009	87	0.0001	0.8	0.9016
II (*Unio crassus* only)	Intercept	0.08	1	0.08	174.7	< 0.0001
	River	0.0007	2	0.0003	0.7	0.4836
	Female length	0.0008	1	0.0008	1.8	0.1922
	River × female length	0.001	2	0.0005	1.1	0.3565
	WSE	0.0001	29	0.000003	0.1	1.0
	WSE × river	0.0004	58	0.000008	0.3	1.0
	WSE × female length	0.00008	29	0.000003	0.1	1.0
	WSE × river × female length	0.0005	58	0.000009	0.3	1.0

There was no significant difference in the glochidia length of *U. crassus* between the three populations inhabiting different rivers (Table 2).

Discriminant Function Analysis showed that glochidium length had a high discriminant power (Wilk's λ = 0.009; F = 4541.9; df = 3; p < 0.0001) and may be used in species classification. Obtained classification functions were: *a* = 5814.9, *c* = − 1196.0 in *A. anatina*; *a* = 4647.4, *c* = − 764.5 in *A. cygnea*; *a* = 2877.6, *c* = − 294.0 in *U. crassus*; *a* = 2968.2, and *c* = − 313.0 in *U. tumidus*, where *a* is a regression coefficient and *c* is a constant. The classification matrix showed that overall 93.6% of cases were classified correctly (100% of *A. anatina* cases, 100% of *A. cygnea* cases, 80.6% of *U. crassus* cases and 91.7% of *U. tumidus* cases).

### Glochidia shape analyses

The analysis of glochidia shape showed that the first four PCs obtained from Elliptical Fourier Descriptors explained 90.8% of the variation in glochidia shape (PC1: 57.2%; PC2: 22.3%; PC3: 7.9%; PC4: 3.4%) and thus were selected for further analyses. The shape aspect described by each PC was presented at Fig. 1b.

**Figure 3:**
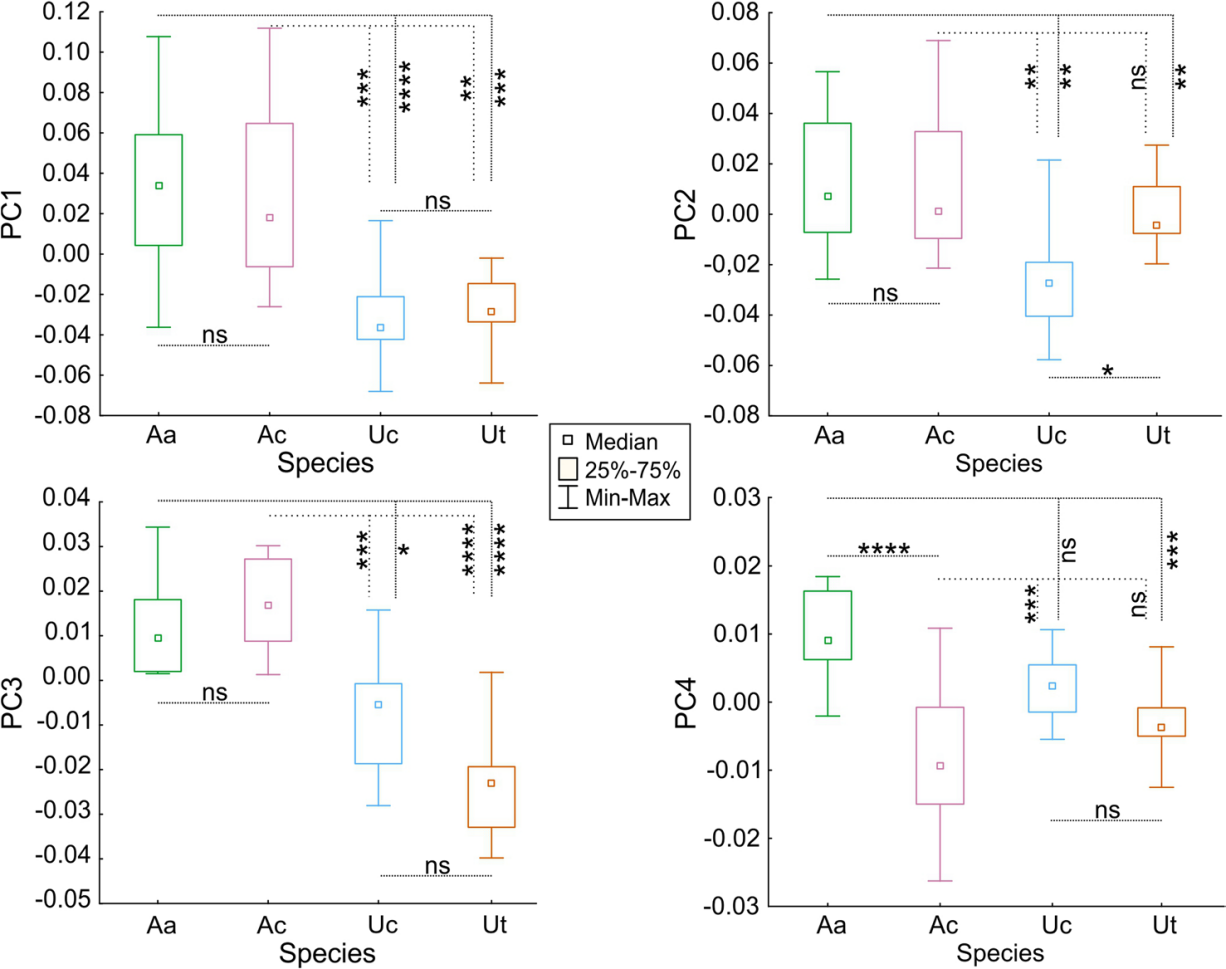
Box plots of the differences in Principal Component scores between species, describing their glochidia shape obtained from Elliptical Fourier Descriptors. Aa, *Anodonta anatina*; Ac, *Anodonta cygnea*; Uc, *Unio crassus*; Ut, *Unio tumidus*; ns, not significant; *p < 0.05, **p < 0.01, ***p < 0.001, ****p < 0.0001.

The differences in PC scores between species were significant for all PCs: PC1 (the symmetry vs vertical axis; Kruskal–Wallis test: H = 35.0; df = 3; p < 0.0001), PC2 (triangular vs hexagonal shape; Kruskal–Wallis test: H = 16.31; df = 3; p = 0.001), PC3 (horizontal rectangle vs vertically extended rectangle shape; Kruskal–Wallis test: H = 40.2; df = 3; p < 0.0001) and PC4 (rhombic shape vs more squared shape; Kruskal–Wallis test: H = 33.4; df = 3; p < 0.0001). The Multiple comparisons post-hoc tests showed that in both PC1 and PC3 *Anodonta* species significantly differed from *Unio* species, while the *A. anatina* did not differ significantly from *A. cygnea* and *U. crassus* did not differ significantly from *U. tumidus* (Fig. 3). In PC2. *U. crassus* differed significantly from other species and *A. cygnea* differed significantly from *U. tumidus* (Fig. 3). In PC4 *A.anatina* differed significantly form *A. cygnea* and *U. tumidus*, *A. cygnea* differed significantly from *U. crasus*, while *U. crassus* did not differ significantly from *U. tumidus* (Fig. 3).

The relationship between female length and shape of its glochidia was not significant in any of the analysed mussel species: *A. anatina* (linear regressions; PC1: b = 0.21, p = 0.445; PC2: b = − 0.11, p = 0.701; PC3: b = − 0.29, p = 0.288; PC4: b = − 0.23, p = 0.415); *A. cygnea* (linear regressions; PC1: b = − 0.38, p = 0.158; PC2: b = 0.06, p = 0.835; PC3: b = 0.29, p = 0.302; PC4: b = − 0.07, p = 0.793); *U. crassus* (linear regressions; PC1: b = − 0.32, p = 0.252; PC2: b = 0.04, p = 0.880; PC3: b = − 0.29, p = 0.302; PC4: b = 0.32, p = 0.248) and *U. tumidus* (linear regressions; PC1: b = 0.31, p = 0.167; PC2: b = − 0.41, p = 0.134; PC3: b = − 0.13, p = 0.642; PC4: b = 0.39, p = 0.146).

## Discussion

Glochidia develop within the environment created by the female mussel and her body size (differing according to habitat and age), which might influence the total amount of energy acquired from the habitat and allocated to progeny^51^. Female size will constrain marsupium size which may set further limits on glochidia numbers but not size or shape. Indeed, in our data, high and significant correlations between female shell length and the wet mass of the gravid marsupium confirm the use of female shell length as a proxy for reproductive output (in accordance with^13,47^).

The zero slope of the regression lines of glochidia size against female lengths (i.e. flat reaction norm^52^) in all species suggests that the glochidium size might be determined during egg formation (Fig. 1a) and seems to be constrained towards a narrow optimum. It is especially visible in *Unio* spp.where glochidium size was remarkably consistent across many factors operating at many scales:

(i) considering phylogeny, glochidia size and variation (Table 2) were stable despite the phylogenetic differences, i.e. both of the *Unio* species did not differ significantly in glochidial size (0.01 mm difference) despite the genetic distance between them^3^;
(ii) despite considerable species-specific differences in habitats (e.g. *U. crassus* numerous in fast-flowing, usually mountainous rivers vs *U. tumidus* occurring rather in lowland rivers and lakes) there was no difference in glochidial size between *Unio* species;
(iii) within a single species (*U. crassus*) glochidia size was consistent across different habitats;
(iv) female size did not influence the glochidia size.

The consistent size of glochidia, irrespective of female size, may be explained by the remarkable life history of naiads. Selection to favour the production of many small glochidia confirms the assertion by Ref.^23^ that the likelihood of successful encystment, subsequent metamorphosis and continuation to adulthood of an individual glochidium is very small. Selection in favour of a high propagule number is further indicated by the rapid and synchronous release of glochidia seen in many species^7,53^, suggesting that fish encounter rates may be relatively rare. If the density of larvae in the water column influences infestation probability, then glochidia might be small not only because of energy allocation in their number, not size but also their size might be selected against to be able to pack them more tightly^54^ into the restricted space of the shell cavity.

Lack of glochidia size differences within the genus *Unio* and between different habitats within the same species (*U. crassus*) nicely evoke J.B.S. Haldane’s idea, that there is one ‘best’ genotype, which is the genotype with the highest fitness^55^. The presence of any additional, less fit genotypes would reduce population mean fitness^56^, leading to its decrease. However, that leads to the prediction that other mussel species must have the same optimal glochidial size, which might be accepted within the genus *Unio*, but surely is not true for genus *Anodonta*.

In *Anodonta* spp.we had no populations of the same species from different habitats. The difference between the two analysed *Anodonta* species in glochidial size could be attributed to (i) species-specific constraints, or (ii) habitat-related effects; at the moment these two factors cannot be distinguished because we do not have data for both species from multiple sympatric populations. However, drawing from other published studies it is apparent that glochidia of *A. anatina* are especially variable in size across studies and may attain very large sizes in some locations (Table 1). The atypically large size of *A. anatina* reported in Ref.^28^ was attributed to hypereutrophic waters, although the population also demonstrated the only record of the species brooding glochidia in all four demibranchs (tetrageny), suggesting other factors may also have contributed to this extreme morphology.

While it is plausible that nutrient-rich waters may result in female mussels investing more energy into each glochidium, variability in glochidia size across populations may also reflect specificity towards particular host fishes. The extreme selective pressure on naiads to successfully attach their glochidia to suitable host fishes has resulted in the evolution of complex lures and attractants in the North American Unionidae^29^. *Anodonta* spp. are generalists, occupying a wide range of habitats, using a wide range of host fishes and releasing their glochidia into the water column where they may encyst the gills or fins of passing fish^3^. Selection may therefore favour the retention of plasticity in glochidia size to enable optimisation towards site-specific host fishes. For example, small glochidia may disperse passively for longer distances in riverine systems, while large glochidia may be more visible to host fishes that attempt to eat them after which the glochidia attach to the host’s gills^20^, or large glochidia may settle more quickly on the benthos where they can encounter benthic-feeding hosts^29^. The different fate of floating glochidia may be attributed also to the shape differences of glochidia: although among *Unio* species there are no differences in glochidial size, their shape differs in one of the shape aspects. This may lead to the conclusion that the size is strongly selected towards the optimum common for *Unio* sp., whereas the shape may differ within the size limit.

The differences in glochidium size between *Unio* and *Anodonta* species might be explained based on proximate and ultimate causes. Considering proximate factors, *Unio* species produce several broods in spring^30^ when low food availability and cool temperatures may limit resources that can be invested in reproduction. Under such conditions, investment in small, fast developing glochidia may prove to be optimal. *Anodonta* species, on the other hand, produce only one brood per season during late summer and brood it through a much longer period than *Unio* species^57^, thus facilitating more investment into each glochidium. Considering ultimate explanations, the glochidium is adapted to infesting fish and its size and morphology likely reflect the foraging habits and abundance of the hosts^29^. *Anodonta* species are relative generalists, using at least 23 species of host fishes^3^ while *Unio* species are more specialist, with *U. tumidus* known to use only six host fishes and *U. crassus* 12 species^3^. The broader spectrum of host species used by *Anodonta* may increase the likelihood of glochidia encountering a suitable host, in which case greater parental investment in each glochidium and its species-specific size and shape may be favoured.

We also show that the differences lie not only in the size of the glochidia but also in their shape. Barnhart et al.^29^ considered the shape of glochidia an important factor concerning host infection strategies. Accepting that in *Unio* sp. the trade-off between size and number of progeny is biased towards the “number” extreme, one can speculate, that under this assumption the size of glochidium cannot vary, thus cannot evolve, whereas the shape of the glochidium does not influence the trade-off, the same number of glochidia might be produced despite their different shapes (differences in PC2 for *Unio* sp. and in PC4 for *Anodonta* sp.).

While size of glochidia may provide some tools for taxonomy. Some research (e.g. Refs.^58,59^) suggest that the shape of the glochidium (symmetry, vertical/horizontal elongation) provides useful taxonomic traits, which can be used as a tool in reconstruction of paleoenvironments^35^. Further understanding of the functional significance of glochidia size and shape may assist with future identification of host fishes and may prove especially useful in the case of rare or poorly studied naiads.

In conclusion, our study demonstrates that glochidium size and shape are under very strong selection towards different optima in different ecological and/or taxonomical groups. The highly conserved glochidia size within *U. crassus*, an endangered and declining species, could be indicative of its narrow host range and suggests that recruitment of the species may be linked closely to the population dynamics of its host fishes. We recommend that closer attention is paid to number, size, shape and variability of glochidia, both within and between mussel species, as this could provide important insights into the ecology and conservation of these important but vulnerable ecosystem engineers.
